# 2-[(Diisopropyl­thio­phosphor­yl)amino]­pyridinium tetra­fluoro­borate

**DOI:** 10.1107/S1600536812022295

**Published:** 2012-05-23

**Authors:** Christian Holzhacker, Karl Kirchner, Kurt Mereiter

**Affiliations:** a Institute of Applied Synthetic Chemistry, Vienna University of Technology, Getreidemarkt 9/163, A-1060 Vienna, Austria; b Institute of Chemical Technologies and Analytics, Vienna University of Technology, Getreidemarkt 9/164SC, A-1060 Vienna, Austria

## Abstract

The title compound, C_11_H_20_N_2_PS^+^·BF_4_^−^, is a salt of 2-(diisopropyl­thio­phospho­ryl­amino)­pyridine, a chelating bidentate ligand that furnishes an S atom as a soft donor and a pyridine N atom as a hard atom for transition-metal complexation. The title salt crystallizes with two formula units in the asymmetric unit. The two independent cations are protonated at the pyridine N atoms and have the S atoms *syn*-oriented to them so as to form bent intra­molecular N—H⋯S hydrogen bonds, one of which one is bifurcated by involving also an N—H⋯F inter­action. The phospho­ryl­amino NH groups form near linear hydrogen bonds to proximal tetra­fluoro­borate anions. Five weak C—H⋯F and three weak C—H⋯S inter­actions link the constituents into a three-dimensional framework. As a result of the crystal packing, the two cations differ notably in conformation, as can be seen from the S—P—N—C torsion angles of −18.7 (1)° in the first and −35.1 (1)° in the second cation.

## Related literature

For the synthesis of 2-(diisopropyl­thio­phospho­ryl­amino)­pyridine, see: Smith & Sisler (1961 ▶); Bichler *et al.* (2011 ▶). For Cu, Pd, and Fe complexes of 2-(diisopropyl­thio­phospho­ryl­amino)­pyridine, see: Öztopcu *et al.* (2011 ▶); Bichler *et al.* (2011 ▶); Holzhacker *et al.* (2011 ▶). For an review on S-bearing transition-metal catalysts, see: Bayón *et al.* (1999 ▶).
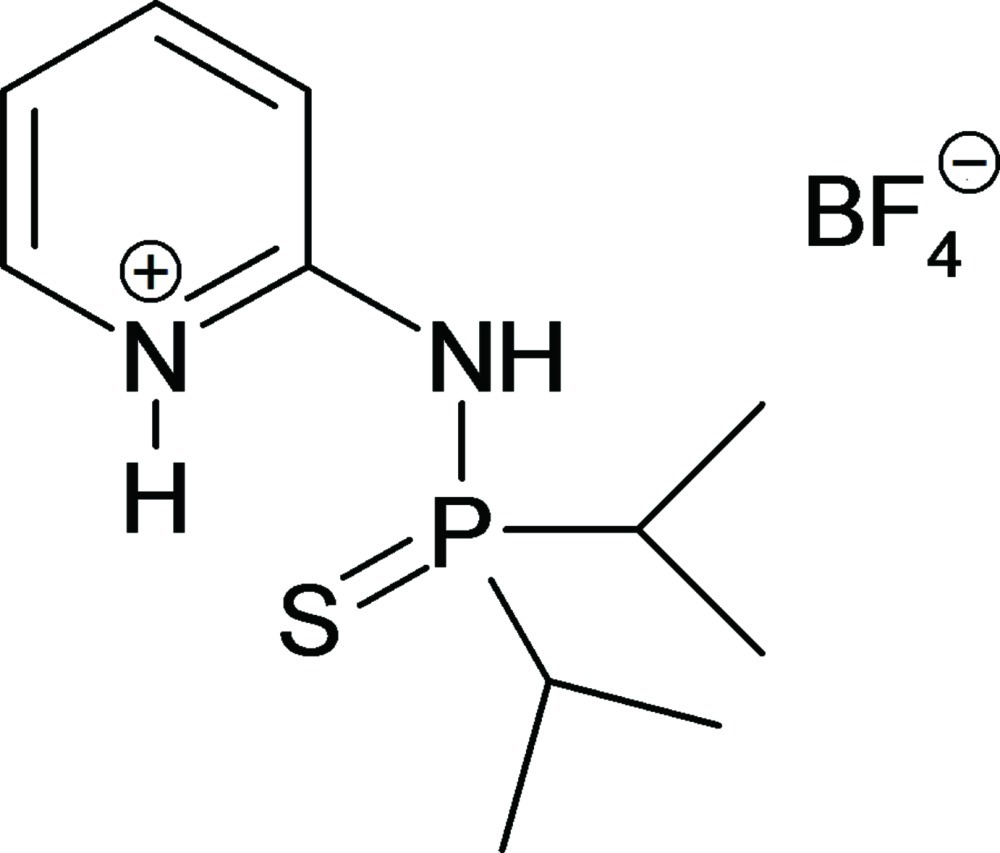


## Experimental

### 

#### Crystal data

C_11_H_20_N_2_PS^+^·BF_4_^−^
*M* *_r_* = 330.13
Triclinic, 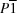

*a* = 9.5816 (1) Å
*b* = 11.3855 (2) Å
*c* = 14.3650 (2) Å
α = 88.221 (1)°
β = 86.067 (1)°
γ = 82.299 (1)°
*V* = 1548.93 (4) Å^3^
*Z* = 4
Mo *K*α radiation
μ = 0.34 mm^−1^
*T* = 100 K
0.54 × 0.40 × 0.38 mm

#### Data collection

Bruker Kappa APEXII CCD diffractometer
Absorption correction: multi-scan (*SADABS*; Bruker, 2008 ▶) *T*_min_ = 0.79, *T*_max_ = 0.86
40158 measured reflections
8997 independent reflections
8332 reflections with *I* > 2σ(*I*)
*R*_int_ = 0.026

#### Refinement

*R*[*F* ^2^ > 2σ(*F* ^2^)] = 0.027
*wR*(*F* ^2^) = 0.076
*S* = 1.02
8997 reflections
385 parameters
H atoms treated by a mixture of independent and constrained refinement
Δρ_max_ = 0.72 e Å^−3^
Δρ_min_ = −0.55 e Å^−3^

### 

Data collection: *APEX2* (Bruker, 2008 ▶); cell refinement: *SAINT* (Bruker, 2008 ▶); data reduction: *SAINT* and *XPREP* (Bruker, 2008 ▶); program(s) used to solve structure: *SHELXS97* (Sheldrick, 2008 ▶); program(s) used to refine structure: *SHELXL97* (Sheldrick, 2008 ▶); molecular graphics: *Mercury* (Macrae *et al.*, 2006 ▶); software used to prepare material for publication: *PLATON* (Spek, 2009 ▶) and *publCIF* (Westrip, 2010 ▶).

## Supplementary Material

Crystal structure: contains datablock(s) I, global. DOI: 10.1107/S1600536812022295/ez2293sup1.cif

Structure factors: contains datablock(s) I. DOI: 10.1107/S1600536812022295/ez2293Isup2.hkl

Supplementary material file. DOI: 10.1107/S1600536812022295/ez2293Isup3.cml

Additional supplementary materials:  crystallographic information; 3D view; checkCIF report

## Figures and Tables

**Table 1:** Hydrogen-bond geometry (Å, °)

*D*—H⋯*A*	*D*—H	H⋯*A*	*D*⋯*A*	*D*—H⋯*A*
N1—H1*N*⋯S1	0.831 (17)	2.482 (16)	3.1356 (9)	136.3 (14)
N1—H1*N*⋯F2^i^	0.831 (17)	2.266 (16)	2.8820 (10)	131.2 (14)
N2—H2*N*⋯F1	0.827 (16)	1.982 (17)	2.8073 (10)	175.6 (15)
N3—H3*N*⋯S2	0.862 (16)	2.375 (16)	3.1297 (9)	146.4 (14)
N4—H4*N*⋯F5	0.824 (17)	2.059 (17)	2.8714 (11)	168.7 (15)
C2—H2⋯F4	0.95	2.39	3.3341 (12)	177
C4—H4⋯F7^ii^	0.95	2.36	3.2290 (12)	151
C6—H6⋯S1^iii^	1.00	2.71	3.7038 (10)	172
C13—H13⋯F7	0.95	2.53	3.2968 (13)	138
C14—H14⋯S1^iv^	0.95	2.85	3.5328 (10)	129
C15—H15⋯F3	0.95	2.52	3.2773 (13)	137
C16—H16⋯F6^i^	0.95	2.41	3.3406 (12)	165
C17—H17⋯S2^v^	1.00	2.73	3.7128 (10)	169

Symmetry codes: (i) 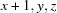; (ii) 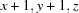; (iii) 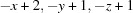; (iv) 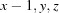; (v) 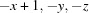.

**Fig. 1.**
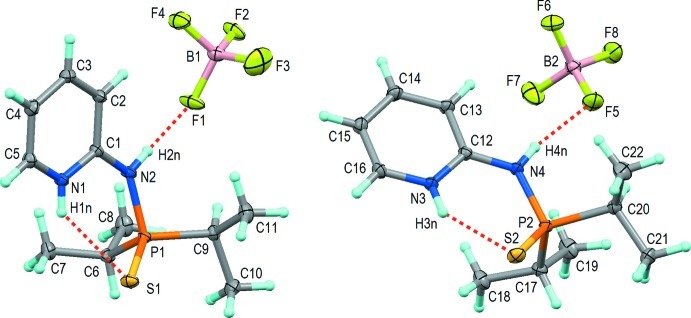
The asymmetric unit of (I) with displacement ellipsoids at the 50% probability level and dashed red lines for hydrogen bonds.

**Fig. 2.**
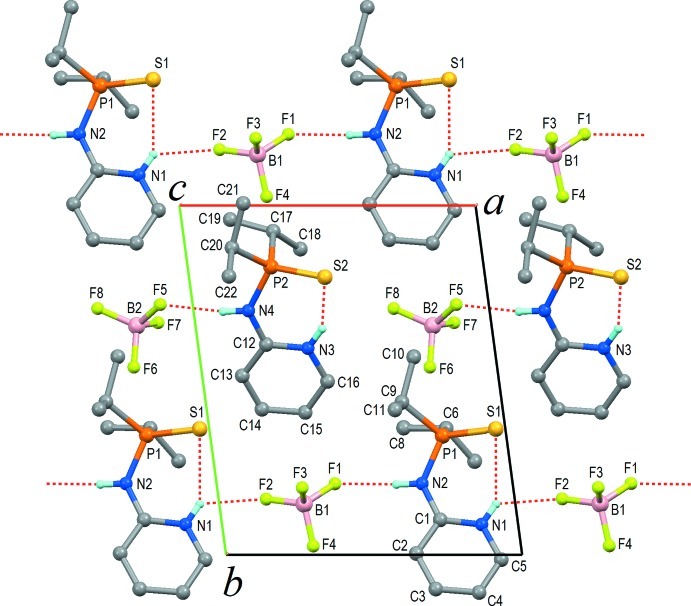
Partial packing diagram of the structure viewed along [001] showing only the N—H···S and N—H···F hydrogen bonds as dashed lines. C-bonded H-atoms omitted for clarity.

## supplementary crystallographic information

### Comment

Dialkyl- and diaryl-thiophosphoryl derivatives of 2-aminopyridine are chelating bidentate ligands, which furnish a pyridine nitrogen as a hard and a sulfur as a soft donor site for transition metal coordination. This dual property is desirable for reactive transition metal complexes (Bayón *et al.*, 1999) and, accordingly, a variety of such complexes have been studied in recent years (Öztopcu *et al.*, 2011; Bichler *et al.*, 2011). During recent work in this field (Holzhacker *et al.*, 2011), the title compound was obtained in suitable crystals and was studied by X-ray diffraction. It crystallizes in the triclinic space group *P*1 and contains two formula units [C_11_H_20_N_2_PS]^+^(BF_4_)^-^ in the asymmetric unit (Fig. 1). In (I), both organocations [*SN-^i^Pr*]H^+^ possess very similar bond lengths and bond angles, but differ significantly in their conformation, as can be seen from the torsion angles S1—P1—N2—C1 = -18.72 (10)° and P1—N2—C1—N1 = -16.91 (14)° in the first cation and corresponding values of -35.14 (10)° and +6.74 (14)° in the second cation. The conformation of the [*SN-^i^Pr*]H^+^ moiety in (I) is related to that of its metal chelate complexes (Öztopcu *et al.*, 2011; Bichler *et al.*, 2011; Holzhacker *et al.*, 2011), which have the S atoms like in (I) *syn*-oriented to the pyridine N, but different from the solid state structure of the unprotonated neutral parent molecule [*SN-^i^Pr*], in which the S atom points away from the pyridine nitrogen corresponding to a S1—P1—N2—C1 torsion angle near 180° (Öztopcu *et al.*, 2011). Compared with the neutral parent molecule [*SN-^i^Pr*], the bond lengths and bond angles in (I) are modestly altered by the pyridine N-protonation with subtle changes of about 0.01 - 0.02 Å in the P1—N2—C1—N1—C5 / P2—N4—C12—N3—C16 system and a significant increase of the ring bond angles C1—N1—C5 / C12—N3—C16 from *ca* 117° in [*SN-^i^Pr*] (Öztopcu *et al.*, 2011) to *ca* 123° in [*SN-^i^Pr*]H^+^ of (I). Both pyridinium moieties in (I) donate strongly bent intramolecular hydrogen bonds to the sulfur atoms as acceptors with N···S distances of 3.136 (1) Å and 3.130 (1) Å for cation 1and 2, respectively (Table 1). For cation 1, the N1—H1n group has in addition to S1 also a fluorine F2(1 + *x*,*y*,*z*) nearby and the entire interaction has to be classified as an asymmetrically branched bifurcated hydrogen bond (N1—H1n···S1,F2; the sum of the three bond angles about H1n is 358°). The phosphorylamine NH groups, which are distinctly acidic irrespective whether the pyridine fragment is protonated or not, form comparatively straight N—H···F hydrogen bonds to the BF_4_ anions with N···F distances of 2.807 (1) and 2.871 (1) Å (Table 1). In the parent compound [*SN-^i^Pr*] the phosphorylamine NH groups donate intermolecular N—H···S hydrogen bonds of N···S = 3.38 and 3.47 Å to neighbouring molecules (Öztopcu *et al.*, 2011). A partial packing diagram showing the N—H donated hydrogen bonds in (I) is presented in Fig. 2. Three-dimensional coherence of the structure is provided by five different C—H···F and three C—H···S interactions listed in Table 1.

### Experimental

2-(Diisopropylthiophosphorylamino)-pyridine (Öztopcu *et al.*, 2011; Smith & Sisler, 1961; 1.03 mmol, 250 mg) was dissolved in dry CH_2_Cl_2_ and 1.05 equivalent of HBF_4_ in Et_2_O was added. The solvent was removed under reduced pressure and the solid residue was washed twice with 15 ml dry Et_2_O. Yield: 326 mg, 95.7%, white solid. Colourless crystals of the title compound for X-ray diffraction were obtained from a CH_2_Cl_2_ solution by vapour diffusion of Et_2_O. ^1^H NMR (δ, CD_2_Cl_2_, 20°C): 15.15 (bs, 1H, [pyH]^+^), 8.12 (t, J = 7.8 Hz, 1H, py^6^),7.98 – 7.90 (m, 2H, py^4^, NH), 7.84 (d, J = 9.0 Hz, 1H, py^3^),7.25 (t, 6.8 Hz, 1H, py^5^), 2.65 – 2.51 (m, 2H, C*H*(CH_3_)_2_), 1.38 – 1.23 (m, 12H, CH(C*H*_3_)_2_). ^13^C{^1^H}NMR (δ, CH_2_Cl_2_, 20°C): 154.6 (s, py^2^), 146.0(s, py^6^), 135.0 (s, py^4^), 118.2 (s, py^5^), 117.2 (s, py^3^), 31.1 (d, J = 57.2 Hz, *C*H(CH_3_)_2_), 15.4 (s, CH(*C*H_3_)_2_), 15.1 (s, CH(*C*H_3_)_2_). ^31^P{^1^H}NMR (δ, CD_2_Cl_2_, 20°C): 107.2.

### Refinement

C-bonded H atoms were placed in calculated positions and thereafter treated as riding, C—H = 0.95–1.00 Å, *U*_iso_(H) = 1.2*U*_eq_(C). N-bonded H atoms were fully refined.

### Crystal data

**Table:**

C_11_H_20_N_2_PS^+^·BF_4_^−^	*Z* = 4
*M**_r_* = 330.13	*F*(000) = 688
Triclinic, *P*1	*D*_x_ = 1.416 Mg m^−^^3^
*a* = 9.5816 (1) Å	Mo *K*α radiation, λ = 0.71073 Å
*b* = 11.3855 (2) Å	Cell parameters from 9086 reflections
*c* = 14.3650 (2) Å	θ = 2.3–30.5°
α = 88.221 (1)°	µ = 0.34 mm^−^^1^
β = 86.067 (1)°	*T* = 100 K
γ = 82.299 (1)°	Prism, colourless
*V* = 1548.93 (4) Å^3^	0.54 × 0.40 × 0.38 mm

### Data collection

**Table:**

Bruker Kappa APEXII CCD diffractometer	8997 independent reflections
Radiation source: fine-focus sealed tube	8332 reflections with *I* > 2σ(*I*)
Graphite monochromator	*R*_int_ = 0.026
φ and ω scans	θ_max_ = 30.0°, θ_min_ = 2.3°
Absorption correction: multi-scan (*SADABS*; Bruker, 2008)	*h* = −13→13
*T*_min_ = 0.79, *T*_max_ = 0.86	*k* = −16→16
40158 measured reflections	*l* = −20→20

### Refinement

**Table:**

Refinement on *F*^2^	Primary atom site location: structure-invariant direct methods
Least-squares matrix: full	Secondary atom site location: difference Fourier map
*R*[*F*^2^ > 2σ(*F*^2^)] = 0.027	Hydrogen site location: inferred from neighbouring sites
*wR*(*F*^2^) = 0.076	H atoms treated by a mixture of independent and constrained refinement
*S* = 1.02	*w* = 1/[σ^2^(*F*_o_^2^) + (0.0381*P*)^2^ + 0.7013*P*] where *P* = (*F*_o_^2^ + 2*F*_c_^2^)/3
8997 reflections	(Δ/σ)_max_ = 0.001
385 parameters	Δρ_max_ = 0.72 e Å^−^^3^
0 restraints	Δρ_min_ = −0.55 e Å^−^^3^

### Special details

**Table:**

Geometry. All e.s.d.'s (except the e.s.d. in the dihedral angle between two l.s. planes) are estimated using the full covariance matrix. The cell e.s.d.'s are taken into account individually in the estimation of e.s.d.'s in distances, angles and torsion angles; correlations between e.s.d.'s in cell parameters are only used when they are defined by crystal symmetry. An approximate (isotropic) treatment of cell e.s.d.'s is used for estimating e.s.d.'s involving l.s. planes.
Refinement. Refinement of *F*^2^ against ALL reflections. The weighted *R*-factor *wR* and goodness of fit *S* are based on *F*^2^, conventional *R*-factors *R* are based on *F*, with *F* set to zero for negative *F*^2^. The threshold expression of *F*^2^ > σ(*F*^2^) is used only for calculating *R*-factors(gt) *etc*. and is not relevant to the choice of reflections for refinement. *R*-factors based on *F*^2^ are statistically about twice as large as those based on *F*, and *R*- factors based on ALL data will be even larger.

### Fractional atomic coordinates and isotropic or equivalent isotropic displacement parameters (Å^2^)

**Table:**

	*x*	*y*	*z*	*U*_iso_*/*U*_eq_	
P1	0.78596 (2)	0.65777 (2)	0.445334 (16)	0.01166 (5)	
S1	0.96769 (2)	0.63976 (2)	0.374765 (18)	0.01685 (5)	
N1	0.87680 (9)	0.91293 (7)	0.39707 (6)	0.01373 (15)	
H1N	0.9345 (17)	0.8543 (14)	0.4094 (11)	0.026 (4)*	
N2	0.69381 (9)	0.79586 (7)	0.43140 (6)	0.01417 (15)	
H2N	0.6070 (17)	0.7983 (13)	0.4336 (11)	0.023 (4)*	
C1	0.73899 (10)	0.89975 (8)	0.40033 (6)	0.01231 (16)	
C2	0.64302 (10)	0.99596 (8)	0.37087 (7)	0.01522 (17)	
H2	0.5447	0.9905	0.3742	0.018*	
C3	0.69272 (11)	1.09772 (9)	0.33728 (7)	0.01724 (18)	
H3	0.6283	1.1623	0.3163	0.021*	
C4	0.83746 (11)	1.10760 (9)	0.33348 (7)	0.01743 (18)	
H4	0.8720	1.1779	0.3099	0.021*	
C5	0.92708 (10)	1.01375 (9)	0.36444 (7)	0.01606 (17)	
H5	1.0254	1.0187	0.3633	0.019*	
C6	0.80053 (10)	0.63744 (8)	0.57047 (7)	0.01523 (17)	
H6	0.8573	0.5584	0.5809	0.018*	
C7	0.88217 (12)	0.73112 (10)	0.60746 (7)	0.02037 (19)	
H7A	0.8247	0.8090	0.6048	0.031*	
H7B	0.9704	0.7333	0.5690	0.031*	
H7C	0.9036	0.7110	0.6722	0.031*	
C8	0.65750 (12)	0.63545 (11)	0.62449 (7)	0.0227 (2)	
H8A	0.6714	0.6250	0.6913	0.034*	
H8B	0.6124	0.5696	0.6033	0.034*	
H8C	0.5971	0.7104	0.6134	0.034*	
C9	0.66301 (10)	0.56210 (8)	0.40889 (7)	0.01525 (17)	
H9	0.5714	0.5805	0.4465	0.018*	
C10	0.72355 (12)	0.43267 (9)	0.42862 (8)	0.0225 (2)	
H10A	0.6565	0.3803	0.4117	0.034*	
H10B	0.7397	0.4216	0.4951	0.034*	
H10C	0.8131	0.4134	0.3917	0.034*	
C11	0.63660 (12)	0.58331 (11)	0.30551 (8)	0.0232 (2)	
H11A	0.7265	0.5699	0.2681	0.035*	
H11B	0.5927	0.6651	0.2956	0.035*	
H11C	0.5737	0.5285	0.2866	0.035*	
P2	0.28135 (2)	0.17525 (2)	0.010066 (16)	0.01218 (5)	
S2	0.45868 (3)	0.20511 (2)	−0.055680 (18)	0.01840 (6)	
N3	0.36925 (9)	0.40856 (7)	0.08654 (6)	0.01469 (15)	
H3N	0.4264 (17)	0.3527 (14)	0.0599 (11)	0.026 (4)*	
N4	0.18559 (9)	0.30094 (7)	0.05392 (6)	0.01499 (15)	
H4N	0.0991 (18)	0.3036 (14)	0.0574 (11)	0.026 (4)*	
C12	0.23059 (10)	0.39780 (8)	0.09024 (6)	0.01343 (16)	
C13	0.13552 (10)	0.48762 (9)	0.13349 (7)	0.01662 (18)	
H13	0.0371	0.4825	0.1382	0.020*	
C14	0.18682 (11)	0.58298 (9)	0.16883 (7)	0.01770 (18)	
H14	0.1229	0.6438	0.1983	0.021*	
C15	0.33146 (11)	0.59206 (9)	0.16226 (7)	0.01705 (18)	
H15	0.3662	0.6585	0.1864	0.020*	
C16	0.42105 (10)	0.50322 (9)	0.12037 (7)	0.01625 (17)	
H16	0.5197	0.5073	0.1148	0.020*	
C17	0.30506 (11)	0.07372 (9)	0.10941 (7)	0.01614 (17)	
H17	0.3626	−0.0008	0.0863	0.019*	
C18	0.38861 (12)	0.12465 (11)	0.18215 (8)	0.0233 (2)	
H18A	0.3314	0.1939	0.2104	0.035*	
H18B	0.4757	0.1483	0.1519	0.035*	
H18C	0.4122	0.0643	0.2307	0.035*	
C19	0.16526 (12)	0.04013 (11)	0.15352 (8)	0.0239 (2)	
H19A	0.1840	−0.0155	0.2060	0.036*	
H19B	0.1168	0.0028	0.1068	0.036*	
H19C	0.1055	0.1116	0.1760	0.036*	
C20	0.15834 (11)	0.12086 (9)	−0.06363 (7)	0.01632 (17)	
H20	0.0659	0.1196	−0.0270	0.020*	
C21	0.21606 (12)	−0.00593 (10)	−0.09231 (8)	0.0229 (2)	
H21A	0.1512	−0.0352	−0.1333	0.034*	
H21B	0.2249	−0.0573	−0.0364	0.034*	
H21C	0.3089	−0.0063	−0.1255	0.034*	
C22	0.13528 (14)	0.20246 (11)	−0.14931 (8)	0.0268 (2)	
H22A	0.2242	0.2008	−0.1876	0.040*	
H22B	0.1031	0.2836	−0.1292	0.040*	
H22C	0.0638	0.1752	−0.1861	0.040*	
B1	0.28660 (12)	0.85386 (10)	0.39883 (8)	0.0185 (2)	
F1	0.40011 (7)	0.79614 (7)	0.44747 (6)	0.02936 (16)	
F2	0.16085 (7)	0.84002 (7)	0.44969 (5)	0.02751 (15)	
F3	0.28753 (12)	0.80759 (9)	0.31181 (6)	0.0517 (3)	
F4	0.29860 (8)	0.97445 (6)	0.39163 (6)	0.03064 (16)	
B2	−0.19867 (12)	0.34423 (10)	0.11792 (9)	0.0193 (2)	
F5	−0.10985 (7)	0.28359 (7)	0.04786 (6)	0.03054 (16)	
F6	−0.22706 (8)	0.46261 (6)	0.09101 (5)	0.02529 (14)	
F7	−0.12520 (9)	0.33533 (7)	0.19898 (6)	0.03483 (18)	
F8	−0.32269 (8)	0.29481 (7)	0.13303 (7)	0.03629 (19)	

### Atomic displacement parameters (Å^2^)

**Table:**

	*U*^11^	*U*^22^	*U*^33^	*U*^12^	*U*^13^	*U*^23^
P1	0.00889 (10)	0.01168 (10)	0.01423 (10)	−0.00092 (7)	−0.00103 (8)	0.00099 (8)
S1	0.01067 (10)	0.01698 (11)	0.02161 (11)	0.00076 (8)	0.00251 (8)	0.00108 (8)
N1	0.0098 (4)	0.0138 (4)	0.0175 (4)	−0.0012 (3)	−0.0016 (3)	0.0018 (3)
N2	0.0077 (3)	0.0133 (3)	0.0212 (4)	−0.0011 (3)	−0.0006 (3)	0.0034 (3)
C1	0.0104 (4)	0.0136 (4)	0.0130 (4)	−0.0015 (3)	−0.0009 (3)	0.0000 (3)
C2	0.0113 (4)	0.0149 (4)	0.0192 (4)	−0.0002 (3)	−0.0028 (3)	0.0011 (3)
C3	0.0167 (4)	0.0144 (4)	0.0203 (4)	0.0002 (3)	−0.0036 (3)	0.0013 (3)
C4	0.0187 (5)	0.0146 (4)	0.0197 (4)	−0.0048 (3)	−0.0020 (3)	0.0010 (3)
C5	0.0137 (4)	0.0165 (4)	0.0189 (4)	−0.0054 (3)	−0.0008 (3)	0.0002 (3)
C6	0.0152 (4)	0.0150 (4)	0.0156 (4)	−0.0018 (3)	−0.0033 (3)	0.0020 (3)
C7	0.0188 (5)	0.0234 (5)	0.0204 (5)	−0.0058 (4)	−0.0050 (4)	−0.0022 (4)
C8	0.0224 (5)	0.0301 (5)	0.0168 (4)	−0.0102 (4)	0.0016 (4)	0.0003 (4)
C9	0.0134 (4)	0.0155 (4)	0.0175 (4)	−0.0037 (3)	−0.0022 (3)	−0.0005 (3)
C10	0.0230 (5)	0.0148 (4)	0.0306 (5)	−0.0038 (4)	−0.0053 (4)	−0.0009 (4)
C11	0.0247 (5)	0.0281 (5)	0.0188 (5)	−0.0081 (4)	−0.0068 (4)	−0.0007 (4)
P2	0.01002 (11)	0.01213 (10)	0.01402 (10)	−0.00055 (8)	0.00027 (8)	−0.00110 (8)
S2	0.01374 (11)	0.01706 (11)	0.02344 (12)	−0.00190 (8)	0.00638 (9)	−0.00245 (9)
N3	0.0101 (4)	0.0149 (4)	0.0187 (4)	−0.0008 (3)	0.0009 (3)	−0.0029 (3)
N4	0.0087 (4)	0.0140 (4)	0.0221 (4)	−0.0010 (3)	0.0005 (3)	−0.0040 (3)
C12	0.0112 (4)	0.0140 (4)	0.0149 (4)	−0.0013 (3)	−0.0002 (3)	−0.0001 (3)
C13	0.0111 (4)	0.0163 (4)	0.0219 (4)	−0.0009 (3)	0.0021 (3)	−0.0029 (3)
C14	0.0155 (4)	0.0164 (4)	0.0206 (4)	−0.0008 (3)	0.0023 (3)	−0.0037 (3)
C15	0.0168 (4)	0.0169 (4)	0.0180 (4)	−0.0043 (3)	−0.0003 (3)	−0.0027 (3)
C16	0.0125 (4)	0.0180 (4)	0.0188 (4)	−0.0038 (3)	−0.0011 (3)	−0.0010 (3)
C17	0.0152 (4)	0.0162 (4)	0.0167 (4)	−0.0010 (3)	−0.0018 (3)	0.0014 (3)
C18	0.0216 (5)	0.0291 (5)	0.0200 (5)	−0.0042 (4)	−0.0075 (4)	0.0022 (4)
C19	0.0221 (5)	0.0290 (5)	0.0214 (5)	−0.0091 (4)	−0.0004 (4)	0.0065 (4)
C20	0.0152 (4)	0.0172 (4)	0.0166 (4)	−0.0009 (3)	−0.0025 (3)	−0.0030 (3)
C21	0.0235 (5)	0.0197 (5)	0.0256 (5)	−0.0005 (4)	−0.0041 (4)	−0.0084 (4)
C22	0.0315 (6)	0.0278 (5)	0.0212 (5)	−0.0007 (4)	−0.0100 (4)	0.0021 (4)
B1	0.0149 (5)	0.0196 (5)	0.0203 (5)	0.0000 (4)	−0.0001 (4)	0.0008 (4)
F1	0.0104 (3)	0.0298 (4)	0.0461 (4)	0.0001 (2)	−0.0016 (3)	0.0148 (3)
F2	0.0114 (3)	0.0351 (4)	0.0361 (4)	−0.0048 (3)	−0.0026 (3)	0.0074 (3)
F3	0.0758 (7)	0.0500 (5)	0.0258 (4)	0.0059 (5)	0.0004 (4)	−0.0137 (4)
F4	0.0229 (3)	0.0203 (3)	0.0485 (4)	−0.0036 (3)	−0.0036 (3)	0.0086 (3)
B2	0.0127 (5)	0.0187 (5)	0.0265 (5)	−0.0025 (4)	0.0001 (4)	−0.0003 (4)
F5	0.0173 (3)	0.0304 (4)	0.0427 (4)	0.0002 (3)	0.0051 (3)	−0.0121 (3)
F6	0.0251 (3)	0.0212 (3)	0.0275 (3)	0.0022 (2)	0.0010 (3)	0.0046 (3)
F7	0.0384 (4)	0.0365 (4)	0.0310 (4)	−0.0084 (3)	−0.0118 (3)	0.0121 (3)
F8	0.0160 (3)	0.0299 (4)	0.0636 (5)	−0.0087 (3)	0.0073 (3)	−0.0091 (4)

### Geometric parameters (Å, º)

**Table:**

P1—N2	1.7100 (8)	N3—C12	1.3478 (12)
P1—C6	1.8177 (10)	N3—C16	1.3612 (12)
P1—C9	1.8199 (10)	N3—H3N	0.862 (16)
P1—S1	1.9434 (3)	N4—C12	1.3669 (12)
N1—C1	1.3463 (12)	N4—H4N	0.824 (17)
N1—C5	1.3614 (12)	C12—C13	1.4049 (13)
N1—H1N	0.831 (17)	C13—C14	1.3746 (14)
N2—C1	1.3651 (12)	C13—H13	0.9500
N2—H2N	0.827 (16)	C14—C15	1.4001 (14)
C1—C2	1.4069 (12)	C14—H14	0.9500
C2—C3	1.3725 (13)	C15—C16	1.3625 (14)
C2—H2	0.9500	C15—H15	0.9500
C3—C4	1.4035 (14)	C16—H16	0.9500
C3—H3	0.9500	C17—C18	1.5315 (14)
C4—C5	1.3617 (14)	C17—C19	1.5323 (15)
C4—H4	0.9500	C17—H17	1.0000
C5—H5	0.9500	C18—H18A	0.9800
C6—C8	1.5303 (15)	C18—H18B	0.9800
C6—C7	1.5323 (14)	C18—H18C	0.9800
C6—H6	1.0000	C19—H19A	0.9800
C7—H7A	0.9800	C19—H19B	0.9800
C7—H7B	0.9800	C19—H19C	0.9800
C7—H7C	0.9800	C20—C22	1.5290 (15)
C8—H8A	0.9800	C20—C21	1.5334 (14)
C8—H8B	0.9800	C20—H20	1.0000
C8—H8C	0.9800	C21—H21A	0.9800
C9—C11	1.5300 (14)	C21—H21B	0.9800
C9—C10	1.5349 (14)	C21—H21C	0.9800
C9—H9	1.0000	C22—H22A	0.9800
C10—H10A	0.9800	C22—H22B	0.9800
C10—H10B	0.9800	C22—H22C	0.9800
C10—H10C	0.9800	B1—F3	1.3704 (14)
C11—H11A	0.9800	B1—F2	1.3904 (13)
C11—H11B	0.9800	B1—F4	1.3932 (13)
C11—H11C	0.9800	B1—F1	1.4097 (13)
P2—N4	1.7063 (8)	B2—F8	1.3828 (13)
P2—C17	1.8155 (10)	B2—F6	1.3870 (13)
P2—C20	1.8168 (10)	B2—F7	1.3956 (14)
P2—S2	1.9517 (3)	B2—F5	1.4078 (14)
			
N2—P1—C6	105.14 (4)	C12—N3—C16	123.33 (8)
N2—P1—C9	102.21 (4)	C12—N3—H3N	117.0 (10)
C6—P1—C9	108.40 (5)	C16—N3—H3N	119.6 (10)
N2—P1—S1	112.59 (3)	C12—N4—P2	129.67 (7)
C6—P1—S1	113.15 (3)	C12—N4—H4N	113.3 (11)
C9—P1—S1	114.42 (3)	P2—N4—H4N	116.9 (11)
C1—N1—C5	122.89 (8)	N3—C12—N4	120.31 (8)
C1—N1—H1N	118.4 (11)	N3—C12—C13	118.05 (9)
C5—N1—H1N	118.3 (11)	N4—C12—C13	121.63 (9)
C1—N2—P1	130.31 (7)	C14—C13—C12	119.09 (9)
C1—N2—H2N	113.2 (11)	C14—C13—H13	120.5
P1—N2—H2N	115.3 (11)	C12—C13—H13	120.5
N1—C1—N2	120.84 (8)	C13—C14—C15	121.22 (9)
N1—C1—C2	118.15 (8)	C13—C14—H14	119.4
N2—C1—C2	121.00 (8)	C15—C14—H14	119.4
C3—C2—C1	119.39 (9)	C16—C15—C14	118.29 (9)
C3—C2—H2	120.3	C16—C15—H15	120.9
C1—C2—H2	120.3	C14—C15—H15	120.9
C2—C3—C4	120.87 (9)	N3—C16—C15	120.00 (9)
C2—C3—H3	119.6	N3—C16—H16	120.0
C4—C3—H3	119.6	C15—C16—H16	120.0
C5—C4—C3	118.20 (9)	C18—C17—C19	110.96 (9)
C5—C4—H4	120.9	C18—C17—P2	110.62 (7)
C3—C4—H4	120.9	C19—C17—P2	112.78 (7)
N1—C5—C4	120.46 (9)	C18—C17—H17	107.4
N1—C5—H5	119.8	C19—C17—H17	107.4
C4—C5—H5	119.8	P2—C17—H17	107.4
C8—C6—C7	111.65 (8)	C17—C18—H18A	109.5
C8—C6—P1	112.90 (7)	C17—C18—H18B	109.5
C7—C6—P1	110.39 (7)	H18A—C18—H18B	109.5
C8—C6—H6	107.2	C17—C18—H18C	109.5
C7—C6—H6	107.2	H18A—C18—H18C	109.5
P1—C6—H6	107.2	H18B—C18—H18C	109.5
C6—C7—H7A	109.5	C17—C19—H19A	109.5
C6—C7—H7B	109.5	C17—C19—H19B	109.5
H7A—C7—H7B	109.5	H19A—C19—H19B	109.5
C6—C7—H7C	109.5	C17—C19—H19C	109.5
H7A—C7—H7C	109.5	H19A—C19—H19C	109.5
H7B—C7—H7C	109.5	H19B—C19—H19C	109.5
C6—C8—H8A	109.5	C22—C20—C21	111.04 (9)
C6—C8—H8B	109.5	C22—C20—P2	110.40 (7)
H8A—C8—H8B	109.5	C21—C20—P2	109.04 (7)
C6—C8—H8C	109.5	C22—C20—H20	108.8
H8A—C8—H8C	109.5	C21—C20—H20	108.8
H8B—C8—H8C	109.5	P2—C20—H20	108.8
C11—C9—C10	111.19 (9)	C20—C21—H21A	109.5
C11—C9—P1	110.76 (7)	C20—C21—H21B	109.5
C10—C9—P1	108.62 (7)	H21A—C21—H21B	109.5
C11—C9—H9	108.7	C20—C21—H21C	109.5
C10—C9—H9	108.7	H21A—C21—H21C	109.5
P1—C9—H9	108.7	H21B—C21—H21C	109.5
C9—C10—H10A	109.5	C20—C22—H22A	109.5
C9—C10—H10B	109.5	C20—C22—H22B	109.5
H10A—C10—H10B	109.5	H22A—C22—H22B	109.5
C9—C10—H10C	109.5	C20—C22—H22C	109.5
H10A—C10—H10C	109.5	H22A—C22—H22C	109.5
H10B—C10—H10C	109.5	H22B—C22—H22C	109.5
C9—C11—H11A	109.5	F3—B1—F2	110.21 (10)
C9—C11—H11B	109.5	F3—B1—F4	110.25 (10)
H11A—C11—H11B	109.5	F2—B1—F4	108.31 (9)
C9—C11—H11C	109.5	F3—B1—F1	110.41 (10)
H11A—C11—H11C	109.5	F2—B1—F1	108.90 (9)
H11B—C11—H11C	109.5	F4—B1—F1	108.70 (9)
N4—P2—C17	105.30 (4)	F8—B2—F6	110.45 (9)
N4—P2—C20	102.38 (4)	F8—B2—F7	110.28 (10)
C17—P2—C20	108.02 (5)	F6—B2—F7	109.24 (9)
N4—P2—S2	112.71 (3)	F8—B2—F5	110.69 (9)
C17—P2—S2	113.40 (3)	F6—B2—F5	108.88 (9)
C20—P2—S2	114.10 (4)	F7—B2—F5	107.23 (9)
			
C6—P1—N2—C1	104.90 (9)	C17—P2—N4—C12	88.97 (10)
C9—P1—N2—C1	−141.95 (9)	C20—P2—N4—C12	−158.17 (9)
S1—P1—N2—C1	−18.72 (10)	S2—P2—N4—C12	−35.14 (10)
C5—N1—C1—N2	178.31 (9)	C16—N3—C12—N4	179.23 (9)
C5—N1—C1—C2	−1.71 (14)	C16—N3—C12—C13	−1.73 (14)
P1—N2—C1—N1	−16.91 (14)	P2—N4—C12—N3	6.74 (14)
P1—N2—C1—C2	163.11 (8)	P2—N4—C12—C13	−172.27 (8)
N1—C1—C2—C3	2.07 (14)	N3—C12—C13—C14	0.93 (14)
N2—C1—C2—C3	−177.95 (9)	N4—C12—C13—C14	179.96 (9)
C1—C2—C3—C4	−1.09 (15)	C12—C13—C14—C15	0.13 (15)
C2—C3—C4—C5	−0.32 (15)	C13—C14—C15—C16	−0.48 (15)
C1—N1—C5—C4	0.30 (15)	C12—N3—C16—C15	1.40 (15)
C3—C4—C5—N1	0.75 (15)	C14—C15—C16—N3	−0.25 (15)
N2—P1—C6—C8	64.31 (8)	N4—P2—C17—C18	−62.07 (8)
C9—P1—C6—C8	−44.41 (8)	C20—P2—C17—C18	−170.90 (7)
S1—P1—C6—C8	−172.43 (6)	S2—P2—C17—C18	61.61 (8)
N2—P1—C6—C7	−61.44 (8)	N4—P2—C17—C19	62.85 (8)
C9—P1—C6—C7	−170.16 (7)	C20—P2—C17—C19	−45.98 (9)
S1—P1—C6—C7	61.82 (7)	S2—P2—C17—C19	−173.47 (7)
N2—P1—C9—C11	64.39 (8)	N4—P2—C20—C22	69.74 (8)
C6—P1—C9—C11	175.09 (7)	C17—P2—C20—C22	−179.43 (7)
S1—P1—C9—C11	−57.61 (8)	S2—P2—C20—C22	−52.35 (8)
N2—P1—C9—C10	−173.23 (7)	N4—P2—C20—C21	−168.00 (7)
C6—P1—C9—C10	−62.53 (8)	C17—P2—C20—C21	−57.18 (8)
S1—P1—C9—C10	64.77 (8)	S2—P2—C20—C21	69.91 (8)

### Hydrogen-bond geometry (Å, º)

**Table:**

*D*—H···*A*	*D*—H	H···*A*	*D*···*A*	*D*—H···*A*
N1—H1*N*···S1	0.831 (17)	2.482 (16)	3.1356 (9)	136.3 (14)
N1—H1*N*···F2^i^	0.831 (17)	2.266 (16)	2.8820 (10)	131.2 (14)
N2—H2*N*···F1	0.827 (16)	1.982 (17)	2.8073 (10)	175.6 (15)
N3—H3*N*···S2	0.862 (16)	2.375 (16)	3.1297 (9)	146.4 (14)
N4—H4*N*···F5	0.824 (17)	2.059 (17)	2.8714 (11)	168.7 (15)
C2—H2···F4	0.95	2.39	3.3341 (12)	177
C4—H4···F7^ii^	0.95	2.36	3.2290 (12)	151
C6—H6···S1^iii^	1.00	2.71	3.7038 (10)	172
C13—H13···F7	0.95	2.53	3.2968 (13)	138
C14—H14···S1^iv^	0.95	2.85	3.5328 (10)	129
C15—H15···F3	0.95	2.52	3.2773 (13)	137
C16—H16···F6^i^	0.95	2.41	3.3406 (12)	165
C17—H17···S2^v^	1.00	2.73	3.7128 (10)	169

Symmetry codes: (i) *x*+1, *y*, *z*; (ii) *x*+1, *y*+1, *z*; (iii) −*x*+2, −*y*+1, −*z*+1; (iv) *x*−1, *y*, *z*; (v) −*x*+1, −*y*, −*z*.
